# A Fast Elitism Gaussian Estimation of Distribution Algorithm and Application for PID Optimization

**DOI:** 10.1155/2014/597278

**Published:** 2014-04-27

**Authors:** Qingyang Xu, Chengjin Zhang, Li Zhang

**Affiliations:** School of Mechanical, Electrical & Information Engineering, Shandong University, Weihai 264209, China

## Abstract

Estimation of distribution algorithm (EDA) is an intelligent optimization algorithm based on the probability statistics theory. A fast elitism Gaussian estimation of distribution algorithm (FEGEDA) is proposed in this paper. The Gaussian probability model is used to model the solution distribution. The parameters of Gaussian come from the statistical information of the best individuals by fast learning rule. A fast learning rule is used to enhance the efficiency of the algorithm, and an elitism strategy is used to maintain the convergent performance. The performances of the algorithm are examined based upon several benchmarks. In the simulations, a one-dimensional benchmark is used to visualize the optimization process and probability model learning process during the evolution, and several two-dimensional and higher dimensional benchmarks are used to testify the performance of FEGEDA. The experimental results indicate the capability of FEGEDA, especially in the higher dimensional problems, and the FEGEDA exhibits a better performance than some other algorithms and EDAs. Finally, FEGEDA is used in PID controller optimization of PMSM and compared with the classical-PID and GA.

## 1. Introduction

Various optimization problems exist in engineering and academic research, which expect to find the best solution. If the problems are conventional or linear, the common mathematical methods will be effective. However, if the problems are too complicated to the common methods, some heuristic algorithms will be considered. Evolutionary algorithms (EAs) are very popular heuristic optimization techniques in the recent years. EAs are general terms of several optimization algorithms that are inspired by the Darwinian theory of natural evolution. It has the capability of solving the complicated optimization problems with nonlinear, high dimension and non-continuous characteristics. The algorithms search the optimal solution from many possible solutions, and the genetic operators, which simulate the principle of natural genetic evolution, are used to update the individuals. By several iterations, the optimal solution will be obtained, such as the genetic algorithms (GAs) [1], evolution strategies (ES), differential evolution (DE) [2, 3], and the artificial immune system (AIS) [4, 5] and also swarm evolutionary algorithm like particle swarm optimization (PSO) [2, 6, 7]. Although these algorithms have applied success to solve kinds of optimization problems [8], there are some inherent drawbacks. For example, if the building blocks spread all over the solutions, the standard EAs have very poor performance [9]. The recombination operators ether breaks the building blocks frequently or do not mix them effectively.

In recent years, estimation of distribution algorithms (EDAs) have attracted a lot of attention. It was proposed by Miuhlenbein and Paaß [10] and emerged as a generalization of EAs for overcoming some problems of EAs, like building blocks broken, poor performance in high dimensional problems, and the difficulty of modeling the distribution of solutions. Sometimes the gene blocks are built based on simple selection and crossover operators are not effective enough to get optimum solution as the blocks may be broken in EAs [9, 11]. Compared with building blocks in EAs, EDAs do not use the crossover or mutation operator to update individuals [12]. Instead, they extract the global statistical information from the superiority individual of previous iteration and build the distribution probability model of solution for sampling new individuals [13]. It is the main advantages of EDAs compared with EAs that the search process is guided by the probabilistic model with explanatory and transparent characteristics [14, 15]. The algorithms are based on the probabilistic models following two steps: (1) Statistics the information of selected individuals and establish the probability model and (2) generate new population by sampling the probability model. Therefore, the new offspring of EDAs is based on the probability distribution instead of performing recombination of individuals as EAs.

The type of probabilistic models used by EDAs and the methods employed to learn them may vary according to the characteristics of the optimization problem. Therefore, different EDAs have been proposed for discrete and continuous problems. In traditional EDAs, the individuals are encoded with binary strings inheritance from EAs. In the population-based incremental learning (PBIL) algorithm [16], the individuals are encoded as fixed length binary strings. The population of solutions is updated by the probability vector, which is initially set to probability 0.5 for each position of the binary strings. For univariate marginal distribution algorithm (UMDA) [10], the frequencies of values on each position are computed according to the selected individuals, which are then used to generate the new population. The compact genetic algorithm (cGA) [17] updates the population according to the probability vector like the PBIL. However, unlike the PBIL, it modifies the probability vector according to the contribution of individuals.

In case of real-valued problems, there are some approaches to extend EDAs to other domains, such as mapping other domains to the domain of fixed-length binary strings and then mapping the solution back to the problem's original domains, or extend or modify the class of probabilistic models to other domains. This first approach might lead to undesirable limitations and errors on real-coded problems. For the second method, the normal pdf is commonly used in continuous EDAs to represent univariate or multivariate distributions. Therefore, some EDAs based on the Gaussian distribution have been designed. In the stochastic hill-climbing with learning by vectors of normal distributions [18], the population of solutions is modeled by a vector of mean of Gaussian normal distribution *μ*_*i*_ for each variable. The standard deviation *σ* is stored globally and it is the same for all variables. After generating a number of new solutions, the mean *μ*_*i*_ are shifted towards the best solutions and the standard deviation *σ* is reduced to make future exploration more specifically. Various ways of modifying the *σ* parameter have been exploited in [19]. Regularized estimation of distribution algorithms (RegEDA) [20] makes use of regularized model estimation in EDAs for continuous optimization. The regularization techniques can lead to more robust model estimation in EDAs. Continuous Gaussian estimation of distribution algorithm (CGEDA) [14] which is a kind of multivariate EDAs is proposed for real-coded problems. Gaussian data distribution and dependent individuals are two assumptions that are considered in CGEDA. In the algorithm, the joint distribution of promising solutions is used in every dimension of the problem. In literature [21, 22], an estimation of distribution algorithm with Gaussian process based on a subspaces method was proposed, which can reduce the computation of complex problems. A real-coded EDA using multiple probabilistic models (RMM) was proposed [23], which includes multiple types of probabilistic models with different learning rates and diversities. There are also other EDAs, which adopt more involved probability models and mixtures of pdfs. However, the probability models cannot reflect the problem completely, because it is hard to obtain an accurate model. In particular, with the increases of number of variables and the number of mixture components, the optimization results become unreliable [24]. Therefore, we specifically focus on the use of the single normal distribution in this paper, as it is more intuitive to be analyzed. Moreover, the use of single and easy normal pdf will not prevent us from obtaining a better understanding of the exploitation of the solutions. We propose a fast elitism Gaussian EDA (FEGEDA) based on the standard process of EDA. A fast learning rule is used to parameters of pdf learning, and an elitism strategy is used for a better performance. Hence, the increased convergence exhibited in this study is expected.

## 2. The Fast Elitism Gaussian EDA

### 2.1. The Framework of the Algorithm

**Figure 1:**
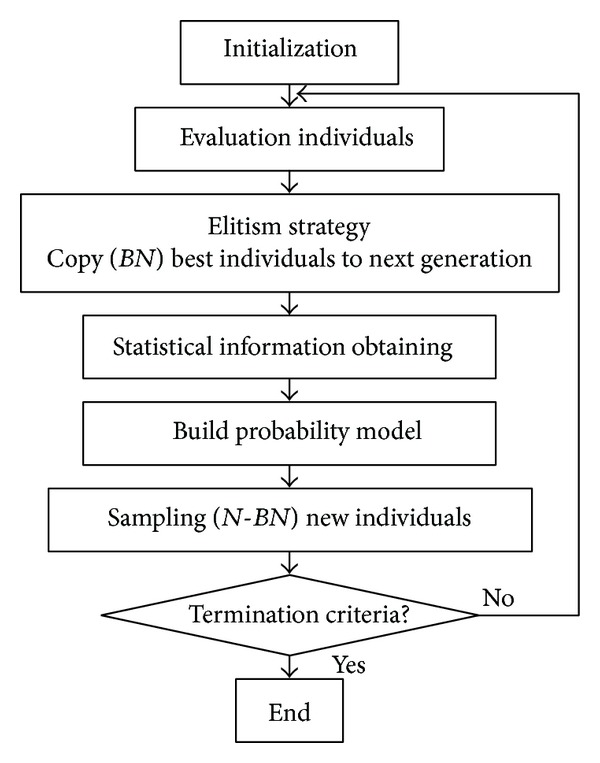
Flowchart of FEGEDA.

EDA is realized by probability estimation and sampling. The probability model is used to estimate the solution distribution, and the probability sampling is used to generate new individuals. In order to improve the performance of standard EDA, we adopt an elitism strategy in FEGEDA.  Figure 1 is the flowchart of FEGEDA.

The steps of the FEGEDA are as follows.

**Step 1 (initialization)**

Set the population size *N*, define the number *BN* of best individuals for probability model establishment and generate the initialized population Pop(0).

**Step 2 (population evaluation)**

Evaluate the *N* individuals *x*_1_, *x*_2_,…*x*_*N*_ according to fitness function *f*(*x*).

**Step 3 (statistical information obtaining)**

Select* BN* best individuals according to the fitness and obtain the statistical information of mean *μ* and standard deviation *σ*.

**Step 4 (probability model *P*(*x*_1_, *x*_2_,…, *x*_*m*_) establishment)**

Use the fast learning rule and build the Gaussian normal distribution by the *u* of means and a covariance *σ*.

**Step 5 (new population Pop(*k*) generation)**

Make use of sampling technique to generate a new population according to the probability model built in Step 4.

**Step 6**

Finally, the iteration is terminated according to the termination criteria. These criteria can be as simple as a fixed number of generations or imply a statistical analysis of the current population to evaluate the stopping condition criteria. If the stopping conditions do not meet, return to Step 2.

The probability model is built according to the distribution of the best solutions in the current population. Therefore, sampling solutions from this model should fall in promising areas with high probability. For some iterations, the solutions should be more likely to be close to the global optimum. The details of the main algorithm are explained in the following.

### 2.2. Initialization

In the algorithm, little parameters are needed to set except for the population size *N* and the best individuals size *BN* selected to build the probability model. Then, a random function is used to generate the initial population according to the variable domain [*L*_*i*_, *H*_*i*_]. Make use of random function generating variables *z*_*i*_ ∈ [*a*_*i*_, *b*_*i*_] and then convert to the domain [*L*_*i*_, *H*_*i*_] by

(1) $$\begin{array}{c} x_{n}^{i}=L_{i}+\frac{H_{i}-L_{i}}{b_{i}-a_{i}}\left(z_{i}-a_{i}\right), \end{array}$$

where *x*_*n*_^*i*^ is the *i*th optimization variable of *n*th individual, *z*_*i*_ is the *i*th random variable, *a*_*i*_ and *b*_*i*_ are the bounds of *i*th random variable, and *L*_*i*_, and *H*_*i*_ are the bounds of *i*th optimization variable.

### 2.3. Population Evaluation

In the individuals' evaluation, it depends on the characteristics of the problem. Conventionally, we should define an objective function *f*(*x*) in order to evaluate the fitness of individuals. Consider

(2) Min⁡(&Max⁡)    y=f(x) S.t.g(x)=[g1(x),g2(x),…,gk(x)]≤0h(x)=[h1(x),h2(x),…,hj(x)]=0x=[x1,x2,…,xi,…,xm]Li≤xi≤Hi (i=1,2,…,m),

where *x* is *m* dimensional optimization variable, *f*(*x*) is the objective function, *g*_*k*_(*x*) is the *k*th inequality constraints, and *h*_*j*_(*x*) is the *j*th equality constraints. *L*_*i*_ and *H*_*i*_ are the bounds of variable.

### 2.4. The Establishment of Probability Model

The most important and crucial step of EDAs is the construction of probabilistic model for the solution distribution; to do this step of FEGEDA, Gaussian distribution of individuals is assumed to model and estimate the distribution of promising solutions in every dimension of the problem. Therefore, mean and standard deviation parameters of promising population are required which computed adaptively by maximum likelihood technique.

#### 2.4.1. Statistical Information Acquisition

In order to construct a pdf model of the promising solutions, we should obtain the statistical information of promising solutions. Hence, statistical techniques have been extensively applied to the optimization problems. Fortunately, these parameters can be efficiently computed by the maximum-likelihood estimations [24].

In the pdf models that assume full independence, every variable is assumed independent of any variable. It must be noted that, in difficult optimization problems, different dependency relations can appear between variables and, hence, considering all of them independent may provide a model that does not represent the problem accurately. However, even if more involved probability models and mixtures of pdfs are defined and used in EDAs, the probability models cannot reflect the problem completely. For system modeling, the dependency relations between variables are very important. Conversely, for optimization problem, the problem decoupled as the combination of some independent variables is expected. Therefore, we specifically focus on the use of independent probability model to construct a fast elitism Gaussian EDA with better performance. That is, the probability distribution *P*(*x*_1_, *x*_2_, …, *x*_*m*_) of the vector (*x*_1_, *x*_2_, …, *x*_*m*_) of *m* variables is assumed to consist of a product of the distributions of individual variables:

(3) $$\begin{array}{c} P\left(x_{1},x_{2},\ldots x_{m}\right)=\overset{m}{\underset{i=1}{\prod }}P\left(x_{i}\right). \end{array}$$

This is very suitable for calculation. Different from the discrete case, the number of parameters to be estimated does not grow exponentially with *m*. Therefore, it is relatively fast and efficient.

The mean and covariance parameters of the normal pdf can be estimated from the selected individuals. Consider

(4) $$\begin{array}{c} {\overset{-}{\mu }}_{i}\left(k\right)=\frac{1}{N}\overset{BN}{\underset{n=1}{\sum }}x_{i}^{n}\left(k\right),\\ \sigma_{i}^{2}\left(k\right)=\frac{1}{N}\overset{BN}{\underset{n=1}{\sum }}\left(x_{i}^{n}\left(k\right)-{\overset{-}{\mu }}_{i}\left(k\right)\right){\left(x_{i}^{n}\left(k\right)-{\overset{-}{\mu }}_{i}\left(k\right)\right)}^{T}, \end{array}$$

${\overset{-}{\mu }}_{i}(k)$ is the mean of *i*th variable in *k*th iteration, *BN* is the selected individuals size, and *σ*_*i*_^2^(*k*) is the covariance of *i*th variable in *k*th iteration.

These parameters are always learned in the process of optimization. The iterative learning approaches are used in some literatures [23, 25–27] as follows:

(5) $$\begin{array}{c} {\overset{-}{\mu }}_{i}\left(k\right)=\alpha {\overset{-}{\mu }}_{i}\left(k\right)+\beta {\overset{-}{\mu }}_{i}\left(k-1\right), \end{array}$$

(6) $$\begin{array}{c} \sigma_{i}^{2}\left(k\right)=\alpha \sigma_{i}^{2}\left(k\right)+\beta \sigma_{i}^{2}\left(k-1\right), \end{array}$$

where *α* and *β* are the weights of ${\overset{-}{\mu }}_{i}(k)$ and ${\overset{-}{\mu }}_{i}(k-1)$. The learning method depends on the class of models used; this step involves parametric or structural learning, also known as model fitting and model selection, respectively. This can improve the performance of EDAs, no matter how simple or complex the learning rule is. We adopt a fast learning method (*α* = 1 and *β* = 0) in this paper, and an elitism strategy is adopted to maintain a smooth convergence process.

#### 2.4.2. Probability Model

In this paper, the normal pdf *N*(*μ*_*i*_, *σ*_*i*_) for variables *x*_*i*_ is parameterized by the *u* of means and covariance *σ*, which is defined by

(7) $$\begin{array}{c} N\left(x_{i},\mu_{i},\sigma_{i}\right)=\frac{1}{\sigma_{i}\sqrt{2\pi }}e^{-{\left(x_{i}-\mu_{i}\right)}^{2}/2\sigma_{i}^{2}}. \end{array}$$

The probability distribution *P*(*x*_1_, *x*_2_,…, *x*_*m*_) of the vector (*x*_1_, *x*_2_,…, *x*_*m*_) of *m* variables is

(8) $$\begin{array}{c} P\left(x_{1},x_{2},\ldots x_{m}\right)=\overset{m}{\underset{i=1}{\prod }}\frac{1}{\sigma_{i}\sqrt{2\pi }}e^{-{\left(x_{i}-\mu_{i}\right)}^{2}/2\sigma_{i}^{2}}. \end{array}$$

The parameters (*μ*_*i*_, *σ*_*i*_) have been estimated according to the selected best individuals. The estimation of marginal parameters (*μ*_*i*_, *σ*_*i*_) is updated in every iteration.

### 2.5. Probability Sampling

The probability sampling is used to generate new individuals using the learned probabilistic models. The sampling method depends on the type of probabilistic model and the characteristics of the problem. For normal pdf problem, a conversion is used in order to convert the normal pdf to a standard normal pdf.

Suppose

(9) $$\begin{array}{c} y=\frac{x-\mu }{\sigma }. \end{array}$$

The normal pdf about *x* is converted to a standard normal pdf about *y*. Consider

(10) $$\begin{array}{c} N\left(x,\mu ,\sigma \right)⟶N\left(y,0,1\right). \end{array}$$

The variable *x* can be calculated by

(11) $$\begin{array}{c} x=\sigma y+\mu . \end{array}$$

In the probability models, every variable (*x*_1_, *x*_2_,…, *x*_*m*_) is assumed independent of any variable. The mean and variance of variable *x*_*i*_ are *μ*_*i*_ and *σ*_*i*_; when *n* → *∞*,

(12) $$\begin{array}{c} y=\frac{\left(\sum_{i=1}^{n}x_{i}-\sum_{i=1}^{n}\mu_{i}\right)}{\sqrt{\sum_{i=1}^{n}\sigma_{i}^{2}}\to N\left(y,0,1\right)}. \end{array}$$

If *x*_*i*_ is the evenly distributed random number of [0, 1],

(13) $$\begin{array}{c} \mu_{i}=E\left(x\right)=\frac{1}{2},\\ \sigma_{i}^{2}=V\left(x\right)=\frac{1}{12}. \end{array}$$

**Figure 2:**
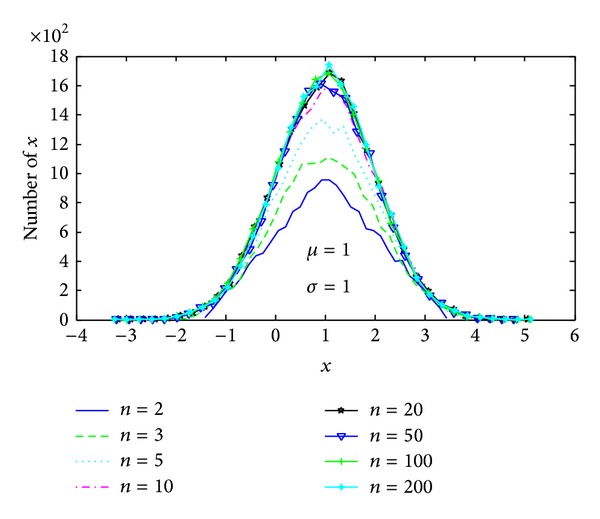
Cartogram of sampling data.

Therefore,

(14) $$\begin{array}{c} y=\frac{\left(\sum_{i=1}^{n}x_{i}-n/2\right)}{\sqrt{n/12}} \end{array}$$

when *n* → *∞* and *y* → *N*(0,1). We can select an appropriate *n* to generate a normal pdf for probability sampling.  Figure 2 shows the cartogram of sampling data in different *n*. From the figure, we can see the sampling data following the pdf better with the increasing of *n*.

### 2.6. Elitism Strategy

**Figure 3:**
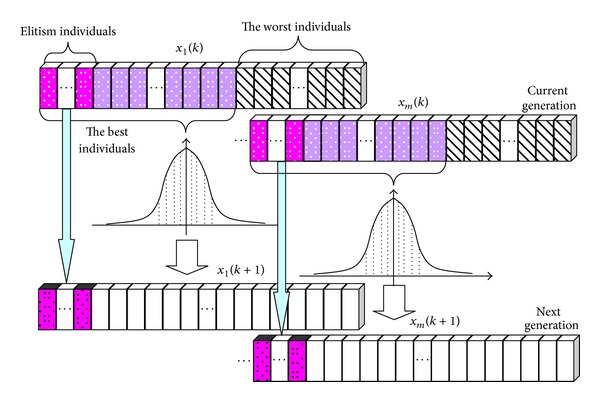
Population operation diagram.

Elitism strategy is an effective strategy to ensure that the best individual is selected as the next generation in EAs, because the best individual may include the information of optimal solution. Therefore, elitism can improve the convergence performance of EAs in many cases [28], and elitism has long been considered an effective method for improving the efficiency of EAs [29]. This is achieved by simply copying the best individual directly to the new generation [30]. However, the number of best individuals selected as the next generation must be handled properly and carefully; otherwise it may lead to premature convergence or cannot improve the efficiency of algorithm.  Figure 3 is the process of new population generation. The elitism individuals will be selected as the new generation directly, and the best individuals are used to establish a probability model to generate other individuals of the next generation. Consider

(15) $$\begin{array}{c} \text{Pop}\left(k\right)=\text{Elitism}{\left(BN\right)}_{k-1}\cup \text{Sample}{\left(N-BN\right)}_{k-1}, \end{array}$$

where Elitism(*BN*) is the operator to copy the best solution of Pop(*k* − 1) to Pop(*k*)  Sample() is the sampling function, *N* is the population size, and *BN* is the best selected individuals number.

## 3. Simulation

In the simulation, in order to exhibit the performance of FEGEDA, we carry out several different simulations, including one-dimensional benchmark, two-dimensional benchmarks, and higher dimensional benchmarks. Moreover, we compare the FEGEDA with other EDAs and other kinds of optimization algorithms.

### 3.1. One-Dimensional Benchmark

One-dimensional problem is easy for FEGEDA. In order to visualize the information of optimization process and models learning process during the evolution clearly, we carry out a one-dimensional benchmark optimization simulation:

(16) $$\begin{array}{c} f_{0}\left(x\right)=\sin\left(x\right)+\sin\left(\frac{10}{3}x\right)+\log\left(x\right)-0.84x+3, \end{array}$$

where *f*_0_ is a multimodal [31], *x* ∈ [2.7,7.5], with several local minimum value, and the global minimum value 1.6013 at *x* = 5.19978.

**Figure 4:**
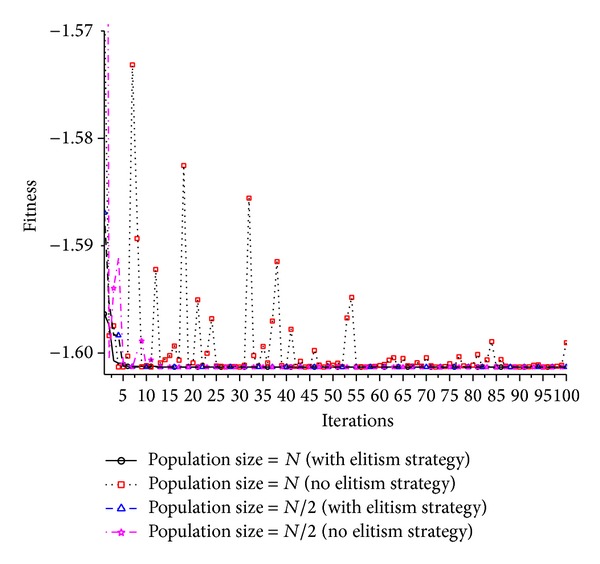
The optimum solutions of each iteration under different conditions.

The best individuals number *BN* selected to build the probability model is a very important parameter for FEGEDA. The elitism strategy is a very important strategy to maintain a smooth optimization process in this paper. Therefore, in order to visualize the performance of corresponding part, we use different BN to testify the effect of outstanding individuals No. to the algorithm performance, and the elitism strategy is optional to testify the effect of the elitism strategy to the convergent performance of the algorithm. Many literatures [32–34] have proved that EDAs are convergent under certain conditions. From Figure 4, we can see that the optimization processes are unstable due to the use of fast learning rule when the algorithm is without elitism strategy no matter what *BN* is. The elitism strategy can make the convergent process smooth and improve the convergent performance too.

**Figure 5:**
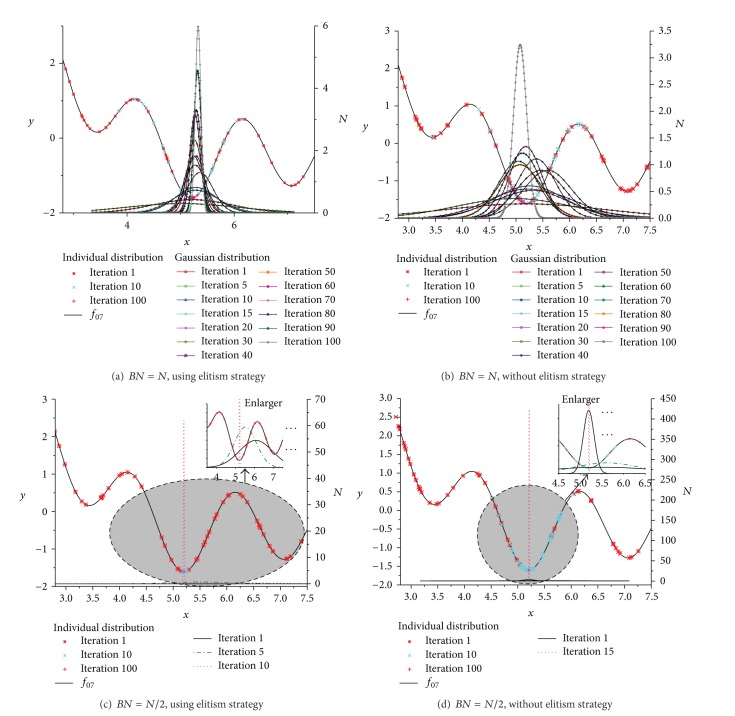
The individuals distribution and probability model of different iterations.

In Figure 5, the individuals' distribution and probability models of some iteration are exhibited. The individuals' distributions of iterations 1, 10, and 100 are shown in the Figure 5. The individuals spread over the optimized function at initial iteration, and then the individuals will focus on the area of optimum solution with the iterations going on. Therefore, the diagram of pdf is flat at the beginning. The parameter *μ* of pdf is smaller and smaller with the increase of iteration and focus on the global optimum solution. The probability models learning process is shown in Figure 5. The elitism strategy is a very important part of the algorithm. Form the exhibition of probability models learning process in Figure 5, we can see that the probability model learning process of solution is smooth when adopting elitism strategy; otherwise it is unstable.

The best selected individuals number is also an important parameter. The convergent speed is faster when the best selected individuals number *BN* is *N*/2. However, if it is too small, it will lead to premature.

**Figure 6:**
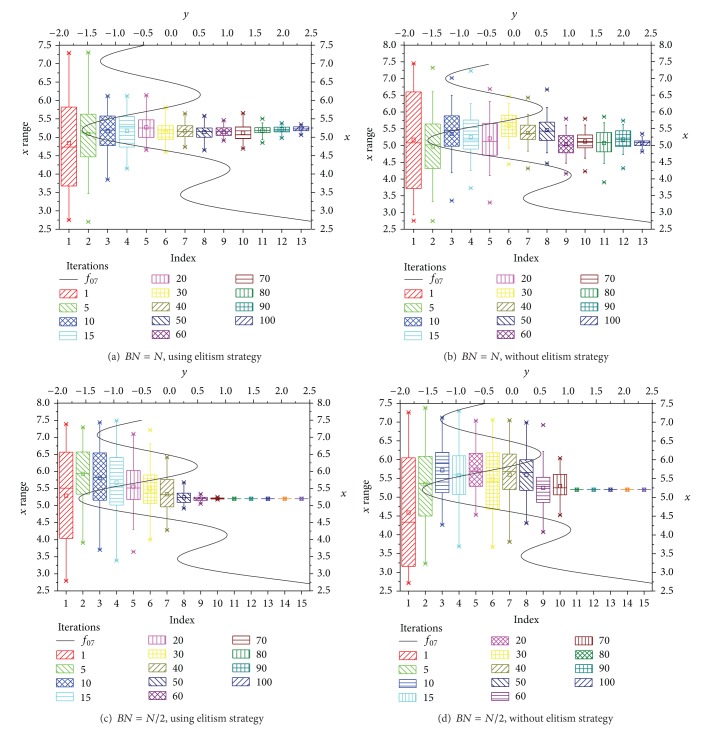
The boxplot of population for different iterations.

Figure 6 is the statistics information of population of some iteration. Form Figure 6, we can see the population distribution when *BN* = *N* using elitism strategy (Figure 6(a)) or without elitism strategy (Figure 6(b)), and *BN* = *N*/2 using elitism strategy (Figure 6(c)) or without elitism strategy (Figure 6(d)). According to Figure 6, the distribution of population is stable when using elitism strategy; otherwise it is fluctuant regardless of *BN* = *N* or *BN* = *N*/2. A small *BN* can make the individuals focus on a certain area quickly.

### 3.2. Two-Dimensional Problems

**Figure 7:**
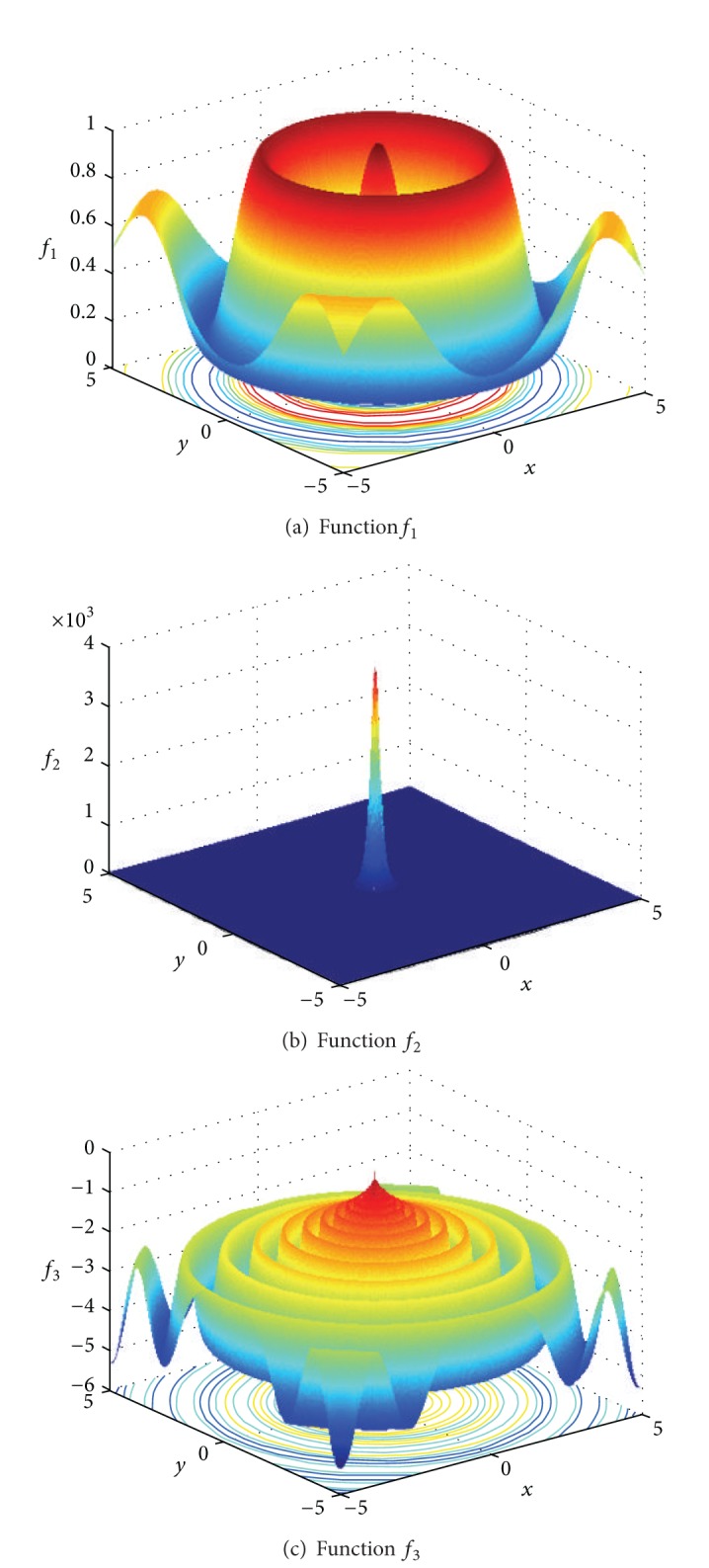
Function diagrams of *f*_1_, *f*_2_, and *f*_3_.

In order to testify the optimization capability of FEGEDA further, three two-dimensional complex functions are considered:

(17) $$\begin{array}{c} f_{1}\left(x,y\right)=0.5-\frac{\text{si}{\text{n}}^{2}\sqrt{x^{2}+y^{2}}-0.5}{{\left(1+0.001*{\left(x^{2}+y^{2}\right)}^{2}\right)}^{2}},\\ f_{2}\left(x,y\right)={\left(\frac{3}{0.05+{\left(x^{2}+y^{2}\right)}^{2}}\right)}^{2}+x^{2}+y^{2}\\ f_{3}\left(x,y\right)=-{\left(x^{2}+y^{2}\right)}^{0.25}\left(\text{si}{\text{n}}^{2}50*{\left(x^{2}+y^{2}\right)}^{0.1}+0.1\right), \end{array}$$

where *x*, *y* ∈ [−5.12, 5.12]. *f*_1_ has infinite maximum value, and the global maximum value 1 is point (0,0). A circuit ridge surrounds the global maximum value. Hence, it is easy to fall into local maximum, which can be used to test the global searching capability of the algorithm. *f*_2_ is a local peak function, and the maximum value is 3600 at point (0,0). This function can be used in determining the local searching capability of the algorithm. The *f*_3_ function is a complicated function with a vibrating circuit ridge outside the global maximum value 0. This function can verify the global and local optimization capability of the algorithm simultaneously.  Figure 7 shows the functions *f*_1_, *f*_2_, and *f*_3_ correspondingly. We compare the optimization result with three other algorithms [35].

**Figure 8:**
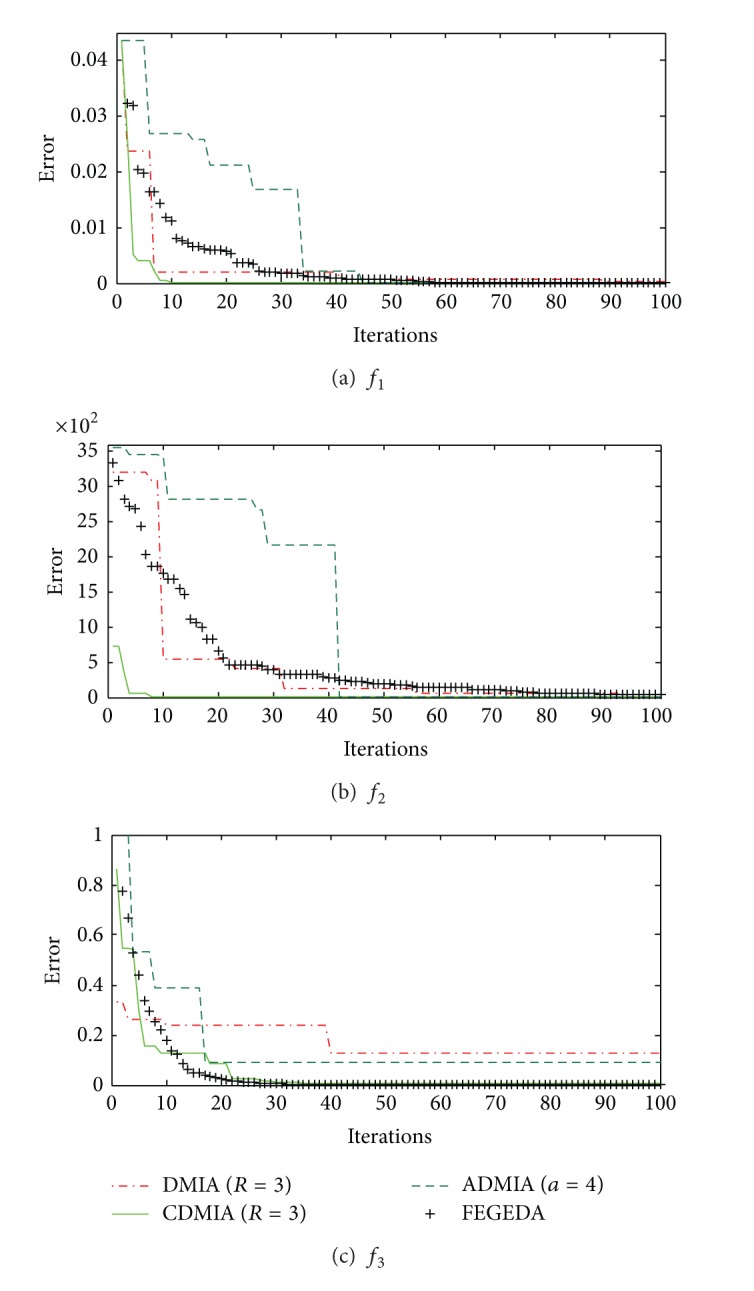
Comparisons of convergent results.

The population size *N* is set to 50, the maximum iteration is set to 100, and *BN* is set to *N*/2 in order to have comparison under the same conditions. From Figure 8, we can see that the FEGEDA can get the optimum solution faster. It has similar optimization capability of CDMIA, which has preferable performance for the three benchmarks.

### 3.3. Higher Dimensional Problems

The advantage of FEGEDA is the capability of higher dimensional problems solution. Some typical benchmarks are considered, including Quadric, Rosenbrock, Ackley, Griewank, Rastrigrin, and Schaffer's *f*_7_ function [21], which are shown in Table 1. In addition, they are configured with a dimension *n* = 10. In order to compare with other EDAs under the same conditions, the population size *N* of FEGEDA is 300 and the maximum iteration is 100.

**Figure 9:**
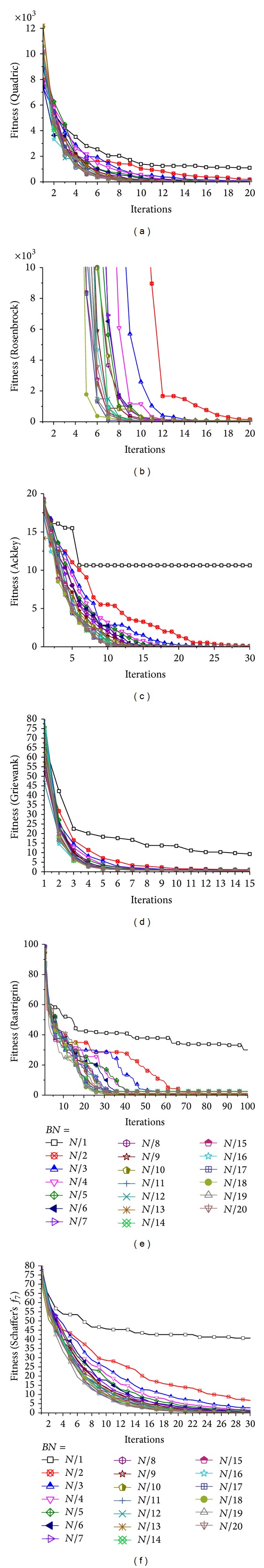
The convergent results under different *BN*.

The algorithm is testified under different *BN* (from *N* to *N*/20). The convergent results under different *BN* are shown in Figure 9. Form Figure 9, we can see that the optimization process is slow when *BN* = *N*. With the decrease of *BN*, the convergent speed is faster. However, the increase of convergent speed is limited. If the *BN* is too small, the optimization will trap into local minimum easily.

**Figure 10:**
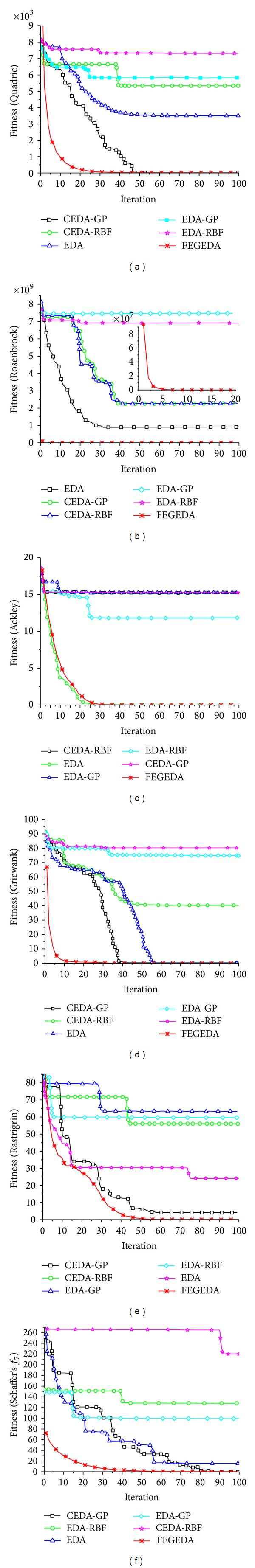
Comparisons of convergent results.

We have a comparison of FEGEDA with other EDAs in [21].  Figure 10 is the comparison diagram. From Figure 10, we can see that FEGEDA is superior to standard EDA and other improved ones for the six benchmarks. For Ackley function, the performance of FEGEDA is the same as EDA. For Rosenbrock function, the initial fitness of FEGEDA is lower than other EDAs. Therefore, we put an enlarger diagram of corresponding area.

## 4. PID Controller Optimization

PID is the most used controller in the permanent magnet synchronous motors (PMSM) control. However, PID controller has poor performance in PMSM control due to the inappropriate parameters. During the past decades, great attention has been paid to the stochastic approach, which is potential in dealing with the problem [36, 37]. In this paper, we adopt FEGEDA to optimize the PID controller of PMSM.

### 4.1. Mathematic Model of PMSM

The mathematical model of PMSM in a *d*, *q* two-phase rotating coordinate system is shown below [38]. The voltage equation is

(18) $$\begin{array}{c} u_{q}=R_{s}i_{q}+L_{q}{\dot{i}}_{q}+\omega_{e}L_{d}i_{d}+\omega_{e}\psi_{f},\\ u_{d}=R_{s}i_{d}+L_{d}{\dot{i}}_{d}-\omega_{e}L_{q}i_{q}, \end{array}$$

where *u*_*d*_ and *u*_*q*_ represent the stator winding shaft in a straight axis and the quadrature voltage, respectively; *i*_*d*_ and *i*_*q*_ are the direct-axis current and quadrature-axis current, respectively; *R*_*s*_ is the stator phase resistance; *L*_*d*_ is the straight axis inductance; *L*_*q*_ is the quadrature axis inductance; *ψ*_*f*_ is the permanent-magnet fundamental excitation magnetic field and stator winding of the magnetic chain; *w*_*e*_ is the electric angular speed of rotor.

The magnetic linkage equation can be expressed as follows:

(19) $$\begin{array}{c} \psi_{d}=L_{d}i_{d}+\psi_{f},\\ \psi_{q}=L_{q}i_{q}, \end{array}$$

where *ψ*_*d*_ and *ψ*_*q*_ represent the syntheses of the magnetic fields in space-direct and quadrature-axis stator winding of the magnetic chain, respectively.

The electromagnetic torque of PMSM in the *d*, *q* coordinate is

(20) $$\begin{array}{c} T_{e}=p_{n}\left(\psi_{f}i_{q}-\left(L_{d}-L_{q}\right)i_{p}i_{d}\right), \end{array}$$

where *p*_*n*_ is number of pole pairs.

According to the motion equation of motor,

(21) $$\begin{array}{c} Jp{\dot{\Omega }}_{r}=T_{e}-T_{l}-B\Omega_{r},\\ \Omega_{r}=\frac{\omega_{e}}{p_{n}}, \end{array}$$

where *Ω*_*r*_ is mechanical angular speed of rotor, *B* is the viscous friction coefficient, *J* is the total moment inertia of rotor and load, and *T*_*l*_ is the load torque.

Thus, the state equation can be derived from the above equations as follows:

(22) $$\begin{array}{c} {\dot{i}}_{q}=\frac{1}{L_{q}}\left(u_{q}-R_{s}i_{q}-L_{d}i_{d}w_{e}-\psi_{f}w_{e}\right),\\ {\dot{i}}_{d}=\frac{u_{d}}{L_{d}}\left(u_{d}-R_{s}i_{d}-w_{e}L_{q}i_{q}\right),\\ {\dot{w}}_{e}=\frac{1.5p_{n}^{2}\left(\psi_{f}i_{q}+\left(L_{d}-L_{q}\right)i_{d}i_{q}\right)-p_{n}T_{m}-Bw_{e}}{J}. \end{array}$$

In the VC system of PMSM, *i*_*d*_ = 0. Therefore, the state space equation (22) is described as

(23) $$\begin{array}{c} {\dot{i}}_{q}=\frac{1}{L_{q}}\left(u_{q}-R_{s}i_{q}-\psi_{f}w_{e}\right),\\ {\dot{w}}_{e}=\frac{1.5p_{n}^{2}\psi_{f}i_{q}-p_{n}T_{m}-Bw_{e}}{J}. \end{array}$$

### 4.2. PID Controller

The continuous form of a PID controller, with input *e* and output *u*, is shown as follows:

(24) $$\begin{array}{c} u\left(t\right)=K_{p}e\left(t\right)+K_{i}\int e\left(t\right)+K_{d}\dot{e}\left(t\right), \end{array}$$

where *K*_*p*_ is the proportional gain, *K*_*i*_ is the integral gains, and *K*_*d*_ is the derivative gains.

There are two types of discrete PID by discretization of continuous PID. The position-type discrete PID is described as

(25) $$\begin{array}{c} u\left(k\right)=K_{p}e\left(k\right)+K_{i}\overset{k}{\underset{j=0}{\sum }}T_{s}e\left(k\right)+\frac{K_{d}}{T_{s}}\left(e\left(k\right)-e\left(k-1\right)\right), \end{array}$$

where *u*(*k*) is the controller output, *e*(*k*) is the error. In practical system control, the integral part is not flexible. Therefore, another velocity-type discrete PID is described as

(26) Δu(k)=KpΔe(k)+KiTse(k)+KdTs(Δe(k)−Δe(k−1)),Δe(k)=e(k)−e(k−1),

where *T*_*s*_ is the sample time. For velocity-type PID, we do not need to calculate the integral part, and the controller output is the increment of PID. Therefore, it is often used in practical system control.

Aggregation function is a conventional method, which can convert a multiobjective problem to a single-objective problem. Consider

(27) $$\begin{array}{c} \text{fitness}=\overset{n}{\underset{i=1}{\sum }}w_{i}f_{i}, \end{array}$$

where fitness is the summation of fitness, *w*_*i*_ is the weight of *i*th objective, and *f*_*i*_ is the fitness value of *i*th objective.

In the optimization process, the objective is to evaluate the performance of PIDs. Thus, for PID, the fitness function is written as [39]

(28) $$\begin{array}{c} f_{1}=\overset{\infty }{\underset{0}{\int }}\left|e\left(t\right)\right|dt\\ f_{2}=\overset{\infty }{\underset{0}{\int }}u^{2}\left(t\right)dt\\ f_{3}=t_{r}, \end{array}$$

where *e*(*t*) is the system error, *u*(*t*) is the control output, and *t*_*r*_ is the rising time.

To avoid overshoot, a penalty value is adopted in the fitness function. That is, once overshoot occurs, the value of overshoot is added to the fitness function. Hence, the penalty function is written as

(29) $$\begin{array}{c} f_{4}=\{\begin{array}{cc} \overset{\infty }{\underset{0}{\int }}\left(y\left(t\right)-y\left(t-1\right)\right)dt & \text{if}\;\;e\left(t\right)<0\\ 0 & \text{if}\;\;e\left(t\right)\geq 0, \end{array} \end{array}$$

where *y*(*t*) is the control output.

Making use of the aggression function, the fitness function is constructed as follows:

(30) $$\begin{array}{c} f=w_{1}f_{1}+w_{2}f_{2}+w_{3}f_{3}+w_{4}f_{4}, \end{array}$$

where *w*_1_, *w*_2_, *w*_3_, and *w*_4_ are the weight coefficients, and *w*_4_ ≫ *w*_1_.

### 4.3. PID Controller Optimization Based on FEGEDA

**Figure 11:**
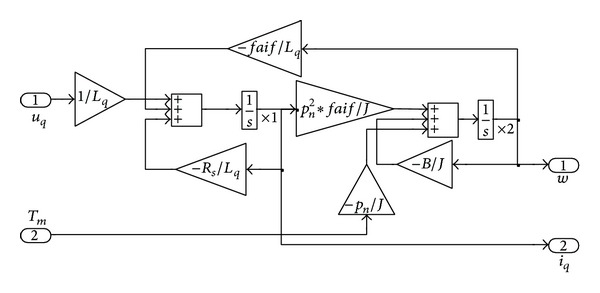
MATLAB/Simulink model of PMSM.

According to state space equation (6), we can build the state space model of PMSM in MATLAB/Simulink as Figure 11. The parameters of PMSM are that *R*_*s*_ is 0.9664, *L*_*q*_ is 0.00621, *P*_*n*_ is 4, *J* is 0.00033, *B* is 0.0001619, and *ψ*_*f*_ is 0.09382 according to motor.

**Figure 12:**
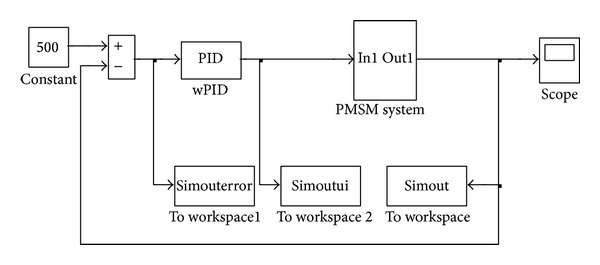
The diagram of PMSM control system.

The component of PMSM is encapsulated into a module. A speed controller added to the speed closed loop.  Figure 12 is the diagram of PMSM control system. The “simouterror,” “simoutui,” and “simout” units are used to record the simulation data for optimization.

**Figure 13:**
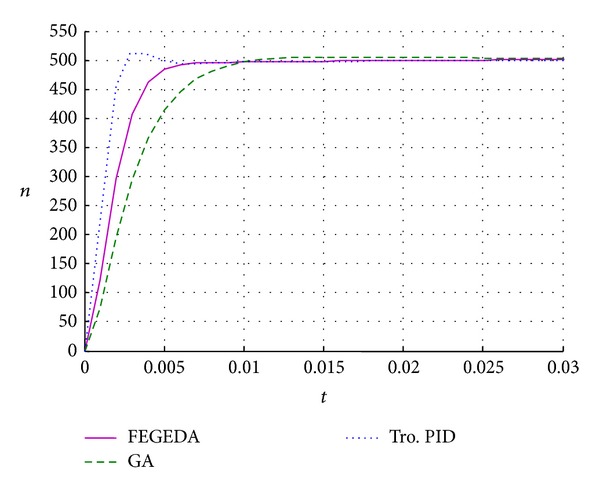
The system response of PMSM with different PIDs.

In order to testify the algorithm, GA and traditional PID are selected to compare against FEGEDA. *w*_1_, *w*_2_, *w*_3_, and *w*_4_ of *f*_*i*_ are set according to the requirement of control system. *w*_1_ is corresponding to the control variable of error, *w*_2_ is a weight coefficient of controlled variable, *w*_3_ is for the control variable of rising time, and *w*_4_ is the penalty of overshoot. If we want a system without overshoot and have a small rising time, *w*_1_, *w*_3_, and *w*_4_ will be set bigger, and *w*_2_ is smaller. If the controlled variable is limited, *w*_2_ will be set bigger. Therefore, these parameters can be set according to the practical requirement. In the test, *w*_1_ is 1, *w*_2_ is 0.1, *w*_3_ is 2, and *w*_4_ is 200. The parameters of GA are that the population size is 30, crossover probability is 0.9, and mutation probability is adaptive to individual fitness. The variable domain of *K*_*p*_ is [0,20] and *K*_*i*_ and *K*_*d*_ are [0,1]. The iteration number is 50. The optimal results are shown in Figure 13. Although the optimal result of traditional PID has shorter response time, the overshoot is bigger. The optimal result of GA has no overshoot, but the system response is slower. Therefore, the performance of FEGEDA is promising.

## 5. Conclusions

We studied the estimation of distribution algorithm in this paper and proposed a fast elitism Gaussian EDA for continuous optimization. Every dimension of individuals is represented by means and standard deviations of Gaussian distribution. These parameters are estimated using maximum likelihood technique by fast learning rule. Then the new population is generated by sampling and elitism strategy. The elitism strategy improves the convergent performance of the algorithm. In the one-dimensional test, we exhibit the optimization process and probability models learning process clearly. In the two-dimensional and higher dimensional problems, we compare the FEGEDA with danger immune algorithm and other EDAs, and the FEGEDA exhibits a good performance. Although the performance of FEGEDA is promising, further studies are still recommended, for instance, how to increase the diversity of population under fast convergence rate.

## Figures and Tables

**Table 1:** High dimensional benchmarks.

Quadric *n* = 10	$f_{4}(x)=\overset{n}{\underset{i=1}{\sum }}{\left(\overset{i}{\underset{j=1}{\sum }}x_{j}\right)}^{2}$, −100 ≤ *x*_*i*_ ≤ 100
Rosenbrock *n* = 10	$f_{5}\left(x\right)=\overset{n-1}{\underset{i=1}{\sum }}\left[100{\left(x_{i+1}-x_{i}^{2}\right)}^{2}+{\left(1-x_{1}\right)}^{2}\right]$, −50 ≤ *x*_*i*_ ≤ 50
Ackley *n* = 10	$f_{6}(x)=-20\exp\left(-0.2\sqrt{\frac{\sum_{i=1}^{n}x_{i}^{2}}{n}}\right)-\exp\left(\sqrt{\frac{\sum_{i=1}^{n}\cos\left(2\pi x_{i}\right)}{n}}\right)+20+e$, −30 ≤ *x*_*i*_ ≤ 30
Griewank *n* = 10	$f_{7}(x)=\frac{1}{4000}\overset{n}{\underset{i=1}{\sum }}{\left(x_{i}\right)}^{2}-\prod_{i=1}^{n}\cos\left(\frac{x_{i}}{\sqrt{i}}\right)+1$, −300 ≤ *x*_*i*_ ≤ 300
Rastrigrin *n* = 10	$f_{8}(x)=\overset{n}{\underset{i=1}{\sum }}\left[x_{i}^{2}-A\cos\left(2\pi x_{i}\right)+A\right]$, −5.12 ≤ *x*_*i*_ ≤ 5.12
Schaffer's *f*_3_ *n* = 10	*f*_9_(*x*) = ∑_*i*=1_^*n*^(*x*_*i*_^2^+*x*_*i*+1_^2^)^0.25^ × {sin⁡⁡[50 × (*x*_*i*_^2^+*x*_*i*+1_^2^)^0.1^] + 1.0}, −100 ≤ *x*_*i*_ ≤ 100
